# Lymph node response to chemoradiotherapy in oesophageal cancer patients: relationship with radiotherapy fields

**DOI:** 10.1007/s10388-020-00777-y

**Published:** 2020-09-05

**Authors:** Willem J. Koemans, Ruben T. H. M. Larue, Maximilian Kloft, Jessica E. Ruisch, Inge Compter, Robert G. Riedl, Lara R. Heij, Wouter van Elmpt, Maaike Berbée, Jeroen Buijsen, Philippe Lambin, Meindert N. Sosef, Heike I. Grabsch

**Affiliations:** 1 Department of Surgery, Zuyderland Medical Center, Heerlen/Sittard, The Netherlands; 2 Department of Pathology, GROW School for Oncology and Developmental Biology, Maastricht University Medical Center+, P. Debyelaan 25, 6229 HX Maastricht, The Netherlands; 3 Department of Surgical Oncology, The Netherlands Cancer Institute, Amsterdam, The Netherlands; 4 Department of Radiation Oncology (MAASTRO), GROW School for Oncology and Developmental Biology, Maastricht University Medical Center+, Maastricht, The Netherlands; 5 The D-Lab, Department of Precision Medicine, GROW School for Oncology and Developmental Biology, Maastricht University Medical Center+, Maastricht, The Netherlands; 6 Department of Pathology, Zuyderland Medical Center, Heerlen/Sittard, The Netherlands; 7 Department of General, Gastrointestinal, Hepatobiliary and Transplant Surgery, RWTH Aachen University Hospital, Aachen, Germany; 8 Department of Pathology, RWTH Aachen University Hospital, Aachen, Germany; 9 Pathology and Data Analytics, Leeds Institute of Medical Research at St James’s, University of Leeds, Leeds, UK

**Keywords:** Oesophageal cancer, Neoadjuvant chemoradiotherapy, Lymph node regression, Radiation field

## Abstract

**Background:**

The presence of lymph node metastasis (LNmets) is a poor prognostic factor in oesophageal cancer (OeC) patients treated with neoadjuvant chemoradiotherapy (nCRT) followed by surgery. Tumour regression grade (TRG) in LNmets has been suggested as a predictor for survival. The aim of this study was to investigate whether TRG in LNmets is related to their location within the radiotherapy (RT) field.

**Methods:**

Histopathological TRG was retrospectively classified in 2565 lymph nodes (LNs) from 117 OeC patients treated with nCRT and surgery as: (A) no tumour, no signs of regression; (B) tumour without regression; (C) viable tumour and regression; and (D) complete response. Multivariate survival analysis was used to investigate the relationship between LN location within the RT field, pathological TRG of the LN and TRG of the primary tumour.

**Results:**

In 63 (54%) patients, viable tumour cells or signs of regression were seen in 264 (10.2%) LNs which were classified as TRG-B (*n* = 56), C (*n* = 104) or D (*n* = 104) LNs. 73% of B, C and D LNs were located within the RT field. There was a trend towards a relationship between LN response and anatomical LN location with respect to the RT field (*p* = 0.052). Multivariate analysis showed that only the presence of LNmets within the RT field with TRG-B is related to poor overall survival.

**Conclusion:**

Patients have the best survival if all LNmets show tumour regression, even if LNmets are located outside the RT field. Response in LNmets to nCRT is heterogeneous which warrants further studies to better understand underlying mechanisms.

**Electronic supplementary material:**

The online version of this article (10.1007/s10388-020-00777-y) contains supplementary material, which is available to authorized users.

## Introduction

The current standard treatment for patients with locally advanced resectable oesophageal cancer (OeC) with curative intent is multimodal therapy, either with neoadjuvant chemoradiotherapy (nCRT) according to the CROSS trial or combination chemotherapy according to the OE02 trial, both followed by surgical resection [1–3]. Although the addition of neoadjuvant treatment offers a survival benefit compared to surgery alone, survival remains poor with 5-year overall survival rates varying between 23 and 47% [3, 4].

A pathological complete response (tumour regression grade [TRG] 1 according to Mandard) of the primary tumour in the resected specimen has been reported in up to 29% of OeC patients treated with nCRT [2, 5]. On the other hand, 38% of OeC patients have limited or no signs of response (TRG 3-5). TRG of the primary tumour has been suggested as a prognostic factor [6]. However, presence of lymph node metastasis (LNmets), their anatomical location and TRG in LNmets (LN-TRG) are potentially better predictors of patient’s survival than TRG of the primary tumour [7–10]. Kadota et al. illustrated the relationship between nCRT response in lymph nodes (LNs) and prognosis in patients with OeC [10]. Furthermore, Philippron et al. showed that even if no viable tumour is found in LNs but LNs show signs of tumour regression, survival is worse compared to patients with truly negative LNs (no evidence of tumour or tumour regression) [11]. These studies suggest that LN-TRG after nCRT and location of LNmets in relation to the radiation field may be important factors for OeC patient’s prognosis. However, previous studies did not evaluate whether there is a relationship between LN location with respect to the radiotherapy (RT) field and LN-TRG.

Elective lymph node irradiation (ENI) and two-field lymphadenectomy are commonly used in OeC patient management to prevent local recurrence and distant metastases. Current ENI guidelines recommend focusing elective irradiation on locoregional LN stations; however, the optimal extent is still under debate [12, 13].

We hypothesized that all LNmets located within the radiotherapy (RT) field will show evidence of tumour regression, confirming current clinical practice [1].

The aim of this study was to investigate the pathological TRG in individual LNmets and relate the results to their location within the RT field in a series of OeC patients treated with nCRT followed by surgical resection.

## Materials and methods

### Patients and treatment

This research has been approved by the Medical Ethical Commission of the Zuyderland Medical Center (Heerlen, NL). All patients diagnosed with oesophageal cancer (OeC) between 2010 and 2016 at the Zuyderland Medical Center (Heerlen, The Netherlands) with clinical TNM 7th edition stage IB–IIIC disease treated with neoadjuvant chemoradiotherapy (nCRT) followed by surgery were included in this study. Clinical assessment according to the Dutch OeC guidelines included fluorodeoxyglucose positron emission tomography (FDG PET/CT) scanning [14]. Approximately 6–10 weeks after completion of nCRT, a re-evaluation PET/CT scan was performed to assess radiological response to therapy and rule out presence of distant metastatic disease. Most patients were treated according to the CROSS trial schedule [2]. Depending on primary tumour location and patient performance status either a minimally invasive transhiatal oesophagogastrectomy including a one-field low mediastinal lymph node (LN) dissection, or a minimal invasive transthoracic approach with Ivor-Lewis-type resection and a two-field LN dissection was performed. All patients had an oesophageal reconstruction using the stomach.

### Radiotherapy planning

A respiratory-gated 4D CT scan was used for RT planning purposes and the following regions of interest were delineated using all diagnostic information available: the gross tumour volume (GTV) defined as the macroscopic outline of the primary tumour and any pathological LNs if applicable; the clinical target volume (CTV) defined as the GTV plus a radial margin of 0.5 cm for LNs or 1 cm for primary tumour, a 3 cm margin in proximal and distal direction and inclusion of elective regional LN regions [12]; the planning target volume (PTV) margin added to account for day-to-day variation in patient positioning and breathing movements. RT was planned to ensure that 99% of the PTV received 95% of the nominal dose of 41.4 Gy, while conforming to the dose constraints of the organs-at-risk (e.g., lungs, heart, and spinal cord). Most patients were radiated 5 days per week in fractions of 1.8 Gy, up to a total of 23 fractions. Concurrently, patients received weekly carboplatin (doses titrated to achieve an area under the curve of 2 mg per milliliter per minute) and paclitaxel (50 mg/m^2^ of body-surface area) for 5 weeks [2]. A minority of patients were treated with 28 × 1.8 Gy.

### Pathology

The surgeon marked the anatomical location of individual LN stations with different coloured beads in the resected specimen according to Casson et al. [15, 16]. In the histopathology laboratory, the fatty tissue was dissected per marked LN station and searched for LNs. All LNs were completely embedded separately by LN station. Tissue was processed into paraffin blocks as per standard protocol. Histopathological examination of the Haematoxylin/Eosin (HE) stained slides was performed by experienced gastrointestinal pathologists. TNM classification 7th ed. was used for tumour staging, grade of differentiation was determined according to the WHO criteria, and the regression of the primary tumour was assessed using the tumour regression grading system according to Mandard [5, 17, 18].

**Fig. 1:**
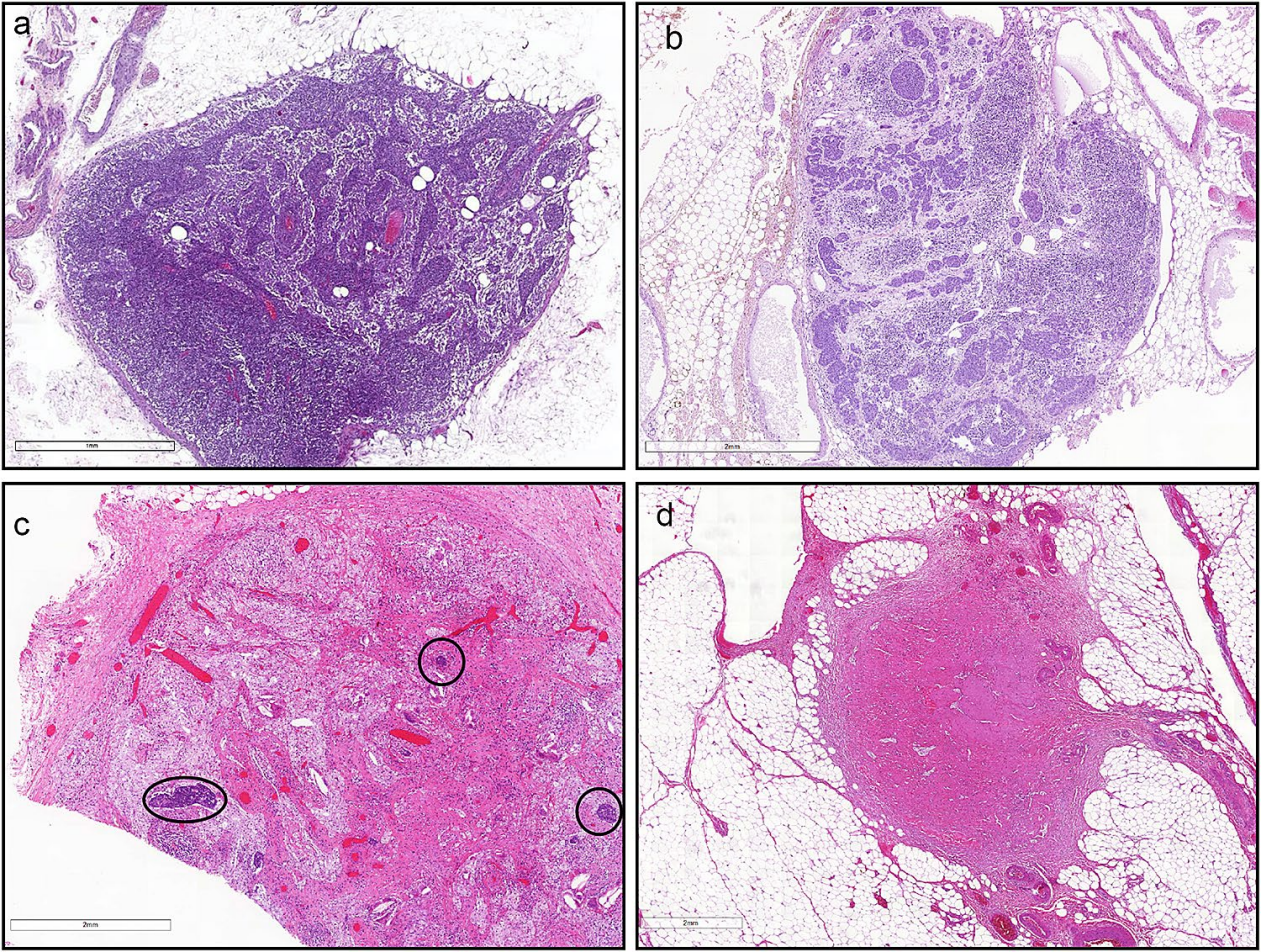
**a** ‘True-negative’ LN without evidence of tumour or tumour regression. **b** LN with viable tumour no regression (class B), **c** LN with viable tumour (circled) and regression (class C), **d** LN with regression no viable tumour (class D)

For the current study, all HE slides from all patients were retrieved retrospectively from the pathology archive and scanned at 20 × magnification using a Panoramic 250 scanner (3DHistech, Budapest, Hungary). Scanned slides with LNs were identified, reviewed and classified according to Martin-Romano et al. by at least two observers as: TRG-A: ‘true-negative’ LN without evidence of tumour or tumour regression; TRG-B: LN with viable tumour without evidence of tumour regression (no fibrosis, no mucin pools); TRG-C: LN with viable tumour and evidence of tumour regression (fibrosis or mucin pools or both); TRG-D: LN without viable tumour and evidence of tumour regression (fibrosis or mucin pools or both) interpreted as ‘complete tumour regression’ [19] (Fig. 1). Patients with only TRG-A LNs were classified as ‘true_ypN0’; while, patients with only TRG-D LNs were classified as ‘complete responders’. All other patients were classified as ‘incomplete responders’ (see Table 1).

**Table 1:** Classification of patients in relation to the tumour regression (TRG) seen in their lymph nodes (LN)

Class	Definition	Classification
A	‘True-negative’ LN without evidence of tumour or tumour regression	true_ypN0
B	LN with viable tumour without evidence of tumour regression (no fibrosis, no mucin pools)	Incomplete responders
C	LN with viable tumour and evidence of tumour regression (fibrosis or mucin pools or both)	Incomplete responders
D	LN without viable tumour and evidence of tumour regression (fibrosis or mucin pools or both)	Complete responders

### Data collection and statistics

Patients were followed up according to standard clinical practice in referring hospitals. Overall survival (OS) time was defined as the time from the last RT fraction to the date of death or last follow-up. Date of death was available from the Dutch population register, and median follow-up time was determined using the ‘inverse Kaplan–Meier’ method [20]. Patient and tumour characteristics (sex, age, tumour location and clinical TNM stage) were retrieved from hospital medical records. The relationship between patient and tumour characteristics and LN-TRG was investigated using the Chi-square test for categorical variables or single-factor ANOVA for continuous variables.

Based on the maps published by Casson et al., the anatomical location of resected LNs was determined using the surgery and histopathology reports [15, 16]. Planning CT scans, RT fields and tumour contours were retrospectively reviewed by two experienced radiation oncologists. Based on anatomical landmarks, individual anatomical LN stations were categorized as being ‘inside’ or ‘outside’ of the RT field/PTV.

Baseline characteristics were compared between the three LN-TRG groups. The relationship between pathological parameters and LN-TRG groups was only analyzed for patients with either TRG-B, TRG-C or TRG-D lymph nodes, thus excluding ‘true_ypN0 patients’ from the analyses. The same cohort was used in the multivariate survival analysis. The relationship between LN location within the RT field (yes/no), pathological TRG of individual LNs, primary tumour TRG and resection margin status was analyzed using multivariate Cox regression analysis. One patient was excluded from the survival analysis due to missing data. P-values less than 0.05 were considered significant.

## Results

In total, 117 patients with clinical stage IB–IIIC oesophageal cancer were included in this study. At the time of analysis, 60 patients were still alive; their median follow-up time was 37 months (95%CI: 29–44 months). Median (range) time between end of nCRT and surgery was 10 weeks (IQR: 9–12 weeks). Clinical characteristics of the patient cohort are provided in Table 2.

**Table 2:** Baseline patient characteristics

Baseline characteristics	True_ypN0 (*n* = 54)	Complete LN responders (*n* = 17)	Incomplete LN responders (*n* = 46)	All (*n* = 117)	*p* values
Age, median (range) years	65 (41–78)	63 (43–75)	65 (47–77)	65 (41–78)	0.588
Gender					
Male	39 (72%)	14 (82%)	41 (89%)	94 (80%)	0.103
Female	15 (28%)	3 (18%)	5 (11%)	23 (20%)	
Histology					
Adenocarcinoma	42 (78%)	15 (88%)	37 (80%)	94 (80%)	0.639
Squamous cell carcinoma	12 (22%)	2 (12%)	9 (20%)	23 (20%)	
Tumour location					
Proximal–middle	9 (17%)	1 (6%)	2 (4%)	12 (10%)	0.055
Distal	23 (43%)	13 (76%)	24 (52%)	60 (51%)	
GEJ–cardia	22 (41%)	3 (18%)	20 (43%)	45 (38%)	
cT-stage					
T1	–	–	1 (2%)	1 (1%)	0.172
T2	14 (26%)	2 (12%)	6 (13%)	22 (19%)	
T3	39 (72%)	14 (82%)	37 (80%)	90 (77%)	
T4	1 (2%)	–	2 (4%)	3 (3%)	
Tx	–	1 (6%)	–	1 (1%)	
cN-stage					
N0	21 (39%)	5 (29%)	3 (7%)	29 (25%)	0.033
N1	16 (30%)	6 (35%)	21 (46%)	43 (37%)	
N2	12 (22%)	3 (18%)	11 (24%)	26 (22%)	
N3	5 (9%)	3 (18%)	10 (22%)	18 (15%)	
Nx	–	–	1 (2%)	1 (1%)	

*cT*-*stage* clinical T-stage, according to 7th edition TNM classification, *cN*-*stage* clinical N-stage, according to 7th edition TNM classification, *GEJ* gastroesophageal junction

### Resected lymph nodes

A total of 2565 LNs was resected, with a median (range) of 20 LNs per patient (6–51 LNs). LNmets were most commonly found in LN stations 17–20 (left gastric artery, common hepatic artery, splenic artery, and celiac axis). For a summary of the frequency of LNs and LNmets per resected LN station, see supplementary table S1. Histopathological characteristics of the resection specimens are available in Table 3.

**Table 3:** Pathological patient characteristics

Baseline characteristics	True_ypN0 (*n* = 54)	Complete LN responders (*n* = 17)	Incomplete LN responders (*n* = 46)	All (*n* = 117)	*p* values*
Pathologic response primary tumour					
TRG 1	14 (26%)	6 (35%)	7 (15%)	27 (23%)	0.056
TRG 2	12 (22%)	2 (12%)	7 (15%)	21 (18%)	
TRG 3	12 (22%)	5 (29%)	9 (20%)	26 (22%)	
TRG 4	11 (20%)	3 (18%)	17 (37%)	31 (26%)	
TRG 5	3 (6%)	–	6 (13%)	9 (8%)	
TRG x	2 (4%)	1 (6%)	0 (%)	3 (3%)	
ypT-stage					
T0	14 (26%)	6 (35%)	7 (15%)	27 (23%)	0.042
T1	9 (17%)	3 (18%)	4 (9%)	16 (14%)	
T2	15 (28%)	5 (29%)	11 (24%)	31 (26%)	
T3	16 (30%)	3 (18%)	24 (52%)	43 (37%)	
ypN-stage					
N0	54 (100%)	17 (100%)	–	71 (61%)	< 0.001
N1	–	–	26 (57%)	26 (22%)	
N2	–	–	12 (26%)	12 (10%)	
N3	–	–	8 (17%)	8 (7%)	
ypTNM vs cTNM stage					
Down	54 (100%)	15 (88%)	23 (50%)	92 (79%)	< 0.001
Same	0 (%)	1 (6%)	17 (37%)	18 (15%)	
Up	0 (%)	0 (%)	5 (11%)	5 (4%)	
N/A	0 (%)	1 (6%)	1 (2%)	2 (2%)	
Circumferential resection margin					
R0	51 (94%)	16 (94%)	39 (85%)	106 (91%)	0.167
R0 (margin < 1 mm)	1 (2%)	0 (%)	3 (7%)	4 (3%)	
R1	1 (2%)	1 (6%)	4 (9%)	6 (5%)	
N/A	1 (2%)	0 (%)	0 (%)	1 (1%)	
Resected LN, *n* median (range)	19 (6–38)	20 (11–38)	21 (7–51)	20 (6–51)	0.693
Resected stations, *n* median (range)	5 (1–9)	6 (2–9)	5 (1–8)	5 (1–9)	0.033

*TRG* tumour regression grade, according to Mandard classification, *ypT-stage* pathological T-stage, according to TNM 7th edition, *ypN-stage* pathological N-stage, according to 7th edition TNM classification, *LN* lymph nodes

*Comparison was done between the groups ‘responders’ and ‘non-responders’

### Relationship between tumour regression in lymph nodes and overall survival

**Fig. 2:**
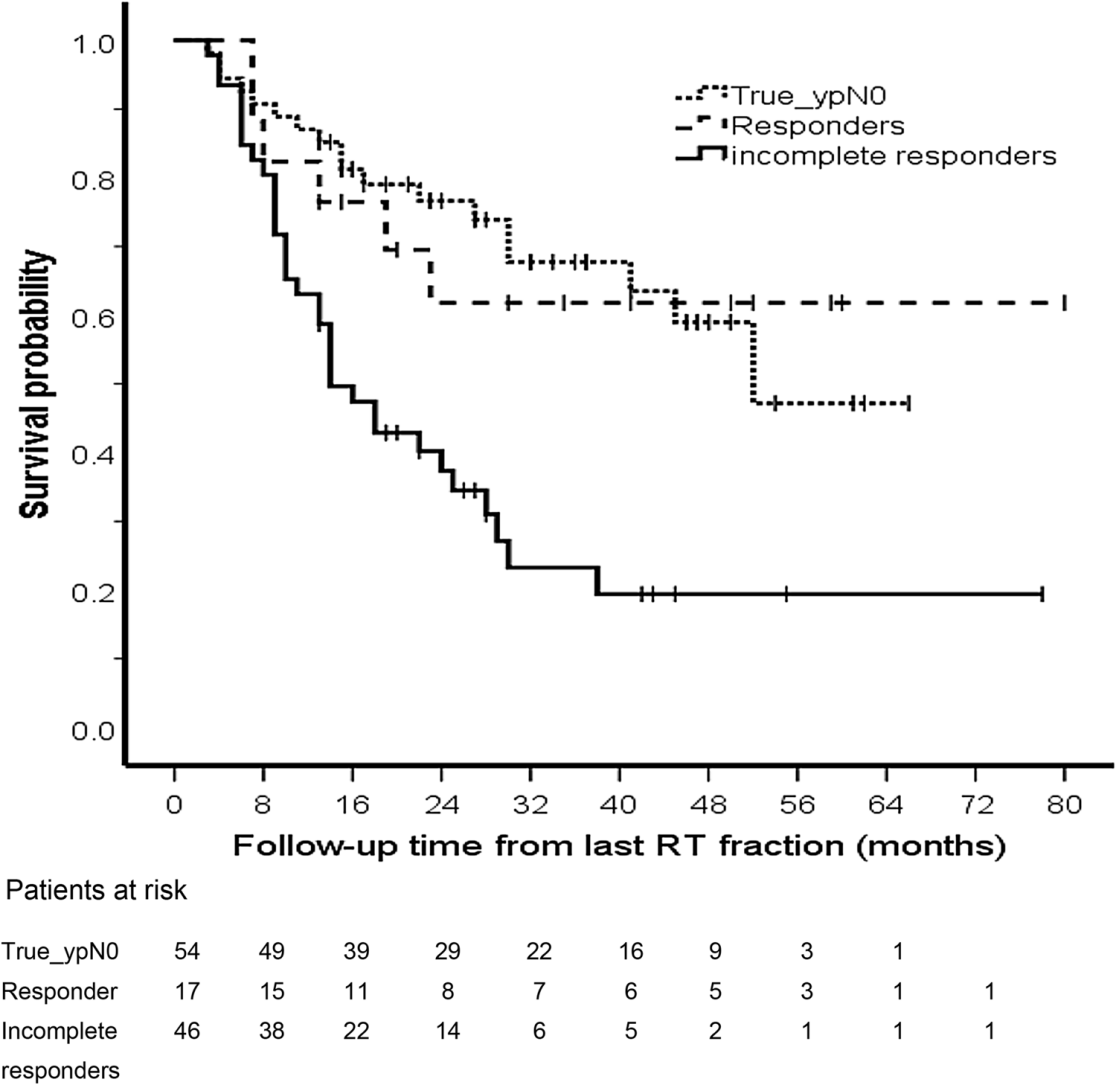
Kaplan–Meier curve showing that overall survival for ypN0 patients and LN responders differs significantly from LN non-responders

In 54 (46%) patients, none of the resected LNs contained tumour or had signs of tumour regression (i.e. all LNs were classified as TRG-A nodes). These patients were classified as ‘true_ypN0’. Overall survival (OS) of the ‘true_ypN0’ patients was significantly longer compared to all other patients (*p* = 0.002), with median overall survival of 52 months and 19 months, respectively, see Fig. 2.

In 63 (54%) patients, a total of 264 LNs contained tumour with or without evidence of regression: 56 (21%) TRG-B, 104 (39%) TRG-C and 104 (39%) TRG-D. 17 (27%) patients were classified as complete LN responders as they had only TRG-D LNs, and 46 (73%) as ‘incomplete LN responders’ as they had vital tumour in at least one LN. There was no difference in OS between true_ypN0 patients (n = 54) and patients in the complete LN responder group (*n* = 17) (Fig. 2, *p* = 0.969). There was a significant difference in OS between ypN + patients and true_ypN0/complete LN responders (Fig. 2, *p* < 0.001, *p* = 0.017, respectively).

### Relationship between clinicopathological data and patients grouped by TRG in lymph nodes

As can be seen in Table 2, baseline clinical characteristics including age, sex, and histology were similar between the patients classified as ‘true_ypN0’, ‘complete LN responders’ or ‘incomplete LN responders’. For the following analyses, ‘true_ypN0’ patients were excluded, leaving 63 patients for analysis. There was a significant relationship between patients classified as ‘complete LN responders’ or ‘incomplete LN responders’ and ypT-stage (*p* = 0.042), number of resected LN stations (*p* = 0.033) and anatomical location of the resected LNs (*p* = 0.035). There was no significant relationship between ‘complete LN responders’ or ‘incomplete LN responders’ and total number of resected LNs (*p* = 0.693) and TRG of the primary tumour (*p* = 0.056).

### Tumour regression in lymph nodes, location within the radiotherapy field and survival

**Fig. 3:**
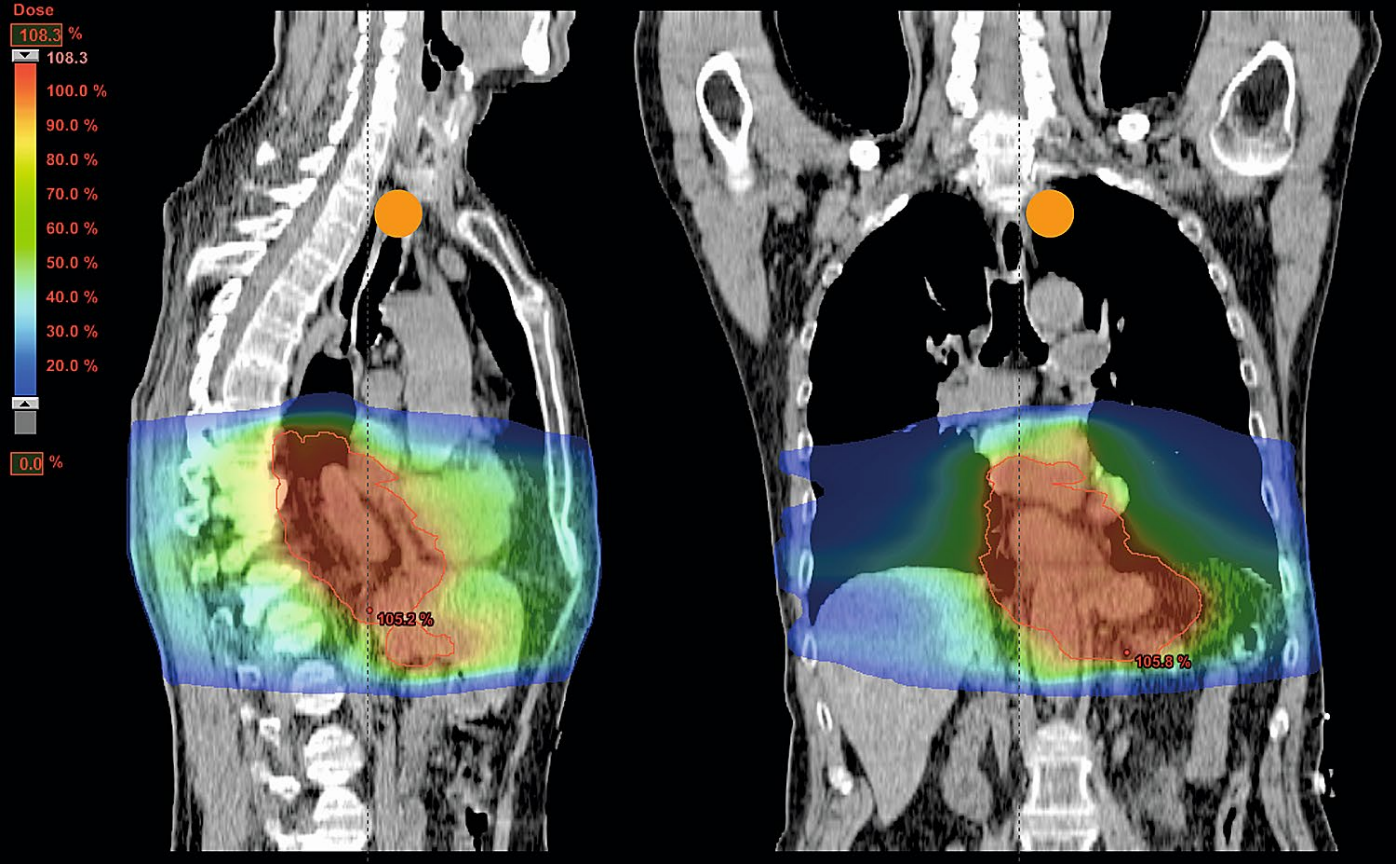
Dose distribution of a representative patient with a cT3N2M0 adenocarcinoma in the gastroesophageal junction. In the resected specimen a lymph node with complete regression was detected in the left upper paratracheal region (2L), indicated in orange

**Fig. 4:**
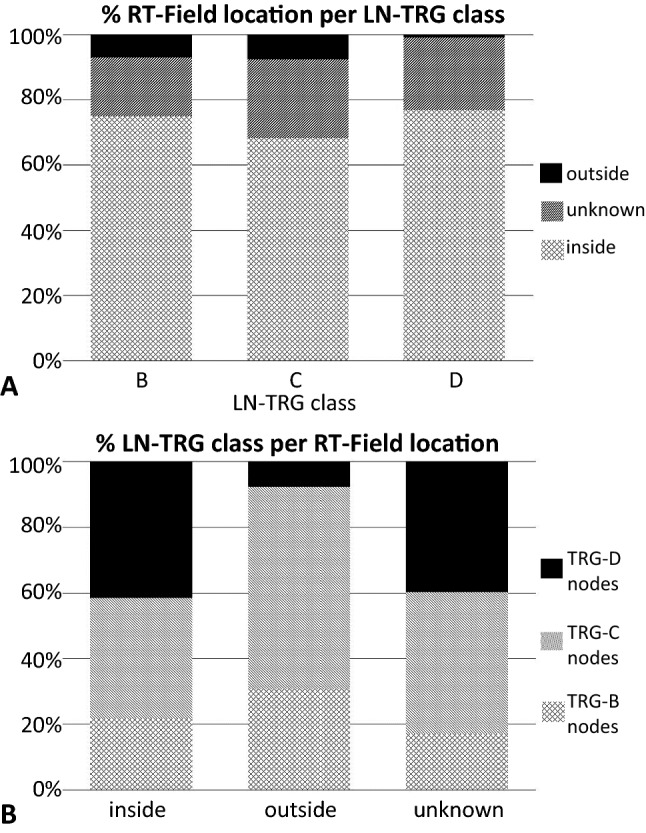
Bar charts showing the distribution of **a** LN TRG with respect to the RT-field location, and **b** LN RT-field location with respect to LN TRG

In depth analysis of the LNs within/outside the RT field focused on the 63 patients with LNmets or LNs with signs of regression, i.e., the ‘complete LN responders’ and ‘incomplete LN responders’. 193 (73%) of the 264 LNmets were found to be located within the planned RT field. 13 of the 264 (5%) LNs from 9 patients were located outside the RT field. Four of these LNs were TRG-B LNs, 8 TRG-C LNs and one TRG-D LN. In 58 LNs (22%), the exact anatomical LN location and, thus, the relation to the RT field could not be ascertained, these were excluded from the analysis. In 7 (78%) of the 9 patients with LNmets outside the RT field, these were located paratracheal/subcarinal with the primary tumour located in the distal oesophagus (2 patients) or at the gastroesophageal junction (5 patients). The other 2 (22%) patients had a primary tumour above the diaphragm with LNmets outside the RT field in the abdominal LN stations. The RT plan of a patient with a LN showing complete regression outside the RT field is illustrated in Fig. 3. There was no significant relationship between LN-TRG and anatomical location of the LN within the RT field (*p* = 0.052, supplementary table S2), probably related to small number of patients. Outside the RT field, 69% of LNmets had signs of tumour regression (Fig. 4).

**Fig. 5:**
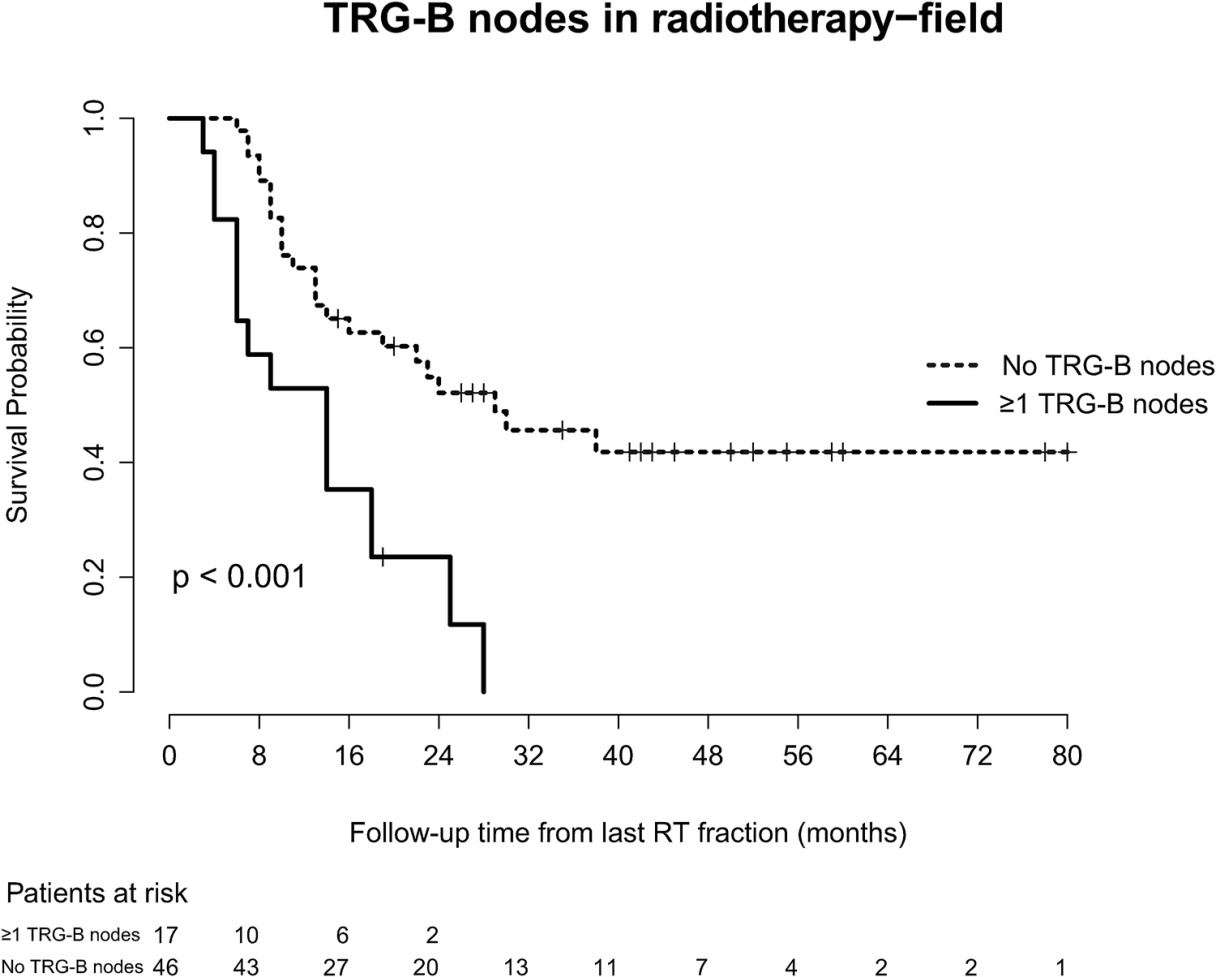
Kaplan–Meier curves for incomplete LN responders, stratified by the number of TRG-B LNs located within the RT-field (no TRG-B nodes versus ≥ 1 TRG-B nodes)

Univariate Cox regression revealed a significant relationship between OS and the number of TRG-B LNs located within the RT field [hazard ratio (HR) 1.1, 95% CI 1–1.3] (table S3). Patients with TRG-B LNs within the RT field had a significantly shorter OS than patients without TRG-B LNs within the RT field, 14 and 29 months, respectively (*p* < 0.001) (Fig. 5).

Univariate analysis comparing TRG of the primary tumour showed that patients with TRG-1 or TRG-2 in the primary tumour had significantly better survival compared to patients with TRG-3, TRG-4 or TRG-5 in the primary tumour (*p* = 0.009).

### Multivariate overall survival analysis

In multivariate analysis, resection margin status (*p* = 0.04) and presence of TRG-B LNs in the RT field (*p* = 0.01) were the only factors significantly related to survival (for details, see Table 4). The regression grade of the primary tumour was not related to survival in multivariate analysis (*p* = 0.69).

**Table 4:** Multivariate overall survival analysis

Factors	Hazard ratio	95% Confidence interval		*p* value
		Lower	Upper	
Resection margin	2.78	1.03	7.51	0.04
TRG primary tumour	0.99	0.97	1.02	0.69
TRG-B LNs in RT field	2.71	1.27	5.78	0.01
TRG-C LNs in RT field	1.58	0.82	3.03	0.17
TRG-D LNs in RT field	1.12	0.55	2.26	0.76
TRG-B LNs outside RT field	2.29	0.64	8.18	0.2
TRG-C LNs outside RT field	0.78	0.21	2.90	0.71
TRG-D LNs outside RT field	7.95	0.82	76.91	0.07

### Heterogeneity in lymph node TRG

Notably, in some patients, a highly heterogeneous LN-TRG was observed. Five (8%) patients had LNs within all three LN-TRG categories (B, C, D) and another 27 (42%) patients had LNs within two of the three categories. In two patients, TRG-D LNs and TRG-B LNs were observed within the same LN station.

## Discussion

Major pathological response in the primary tumour has been related to better overall survival in oesophageal cancer (OeC) patients [6]. However, tumour regression (TRG) in LNmets might be a better predictor of survival than pathological response in the primary tumour [12]. In this study of OeC patients treated with neoadjuvant chemoradiotherapy (nCRT) followed by surgery, we aimed to analyze TRG in LNmets (LN-TRG) in relation to the LN location within the radiotherapy (RT) field and the relationship between LN-TRG and overall survival (OS).

We showed that patients without LNmets and without evidence of LN-TRG, e.g., the ‘true_ypN0’ patients, had a significantly better OS compared to patients with LNmets. Also, patients with evidence of a complete response in their LNs had a better survival than ypN+ patients. Patients in the ‘incomplete responder’ group with one or more LNmets without any sign of tumour regression (TRG-B LNs) had poorer survival than patients in this group with LNmets all showing signs of regression (TRG-C LNs) which was also confirmed in multivariate analysis. These findings support previous findings that LN-TRG might predict OeC patient survival [12]. Our study results suggest that LN-TRG might be related to primary tumour response, a finding which one might expect but to the best of our knowledge has not been reported in the literature. The presence of TRG-B LNs was significantly related to survival in multivariate analysis, when the TRG of the primary tumor was included in the model. Our finding that LN-TRG seems to be a better prognostic factor after chemoradiotherapy than TRG in the primary tumour validates results reported previously by Davies et al. and Urakawa et al. [21, 22].

After nCRT, 40% of OeC patients had evidence of LNmets, which is much lower than at time of diagnosis when 75% of the patients was staged as cN+. This difference can be explained by the complete LN responders (15% of all patients) and the fact that over 60% of the true_ypN0 patients were diagnosed as cN+ prior to treatment suggesting a clinical overdiagnosis of N+ patients. This could be due to false-positive detection in radiological imaging or an incorrect pathological N-stage due to an incomplete lymphadenectomy [23, 24].

We hypothesized that all LNmets within the RT field would show some degree of tumour regression; whereas, this would not apply to LNmets outside the RT field. However, our results do not entirely confirm this hypothesis, as there seems to be a relationship between the location of the LNmets and the regression seen in the LN. But this result is only borderline significant in our study, probably due to small number of patients. The tumour regression seen in LNmets outside the RT field could be explained by the concomitant administered systemic chemotherapy. This chemotherapy will reach the LNs outside the RT field and it is known, from the OE02 trial, that patients treated with chemotherapy have nodal response [11]. Another possibility is that LNs outside the RT field show tumour regression due to a systemic immunological response to the tumour, known as the abscopal effect [25]. However, the fact that LNs of the same patient found within the same LN station can have completely different responses to nCRT was surprising. We hypothesize that this might be due to a difference in blood supply, allowing more chemotherapy to reach the LNmet with the better blood supply. Second, it could be due to tumour clonality or be related to the immune cell population present in the lymph node. It is well known that cancer cells can be highly heterogeneous even within patients [26, 27]. Clonality can be a major factor contributing to resistance against neoadjuvant therapies [28]. Although we have currently no evidence for this, it might be that different clones of the tumour with different resistance patterns populated the LNs located within the same LN station.

Our study has some limitations. As treatment of OeC patients changed with the publication of the CROSS trial results, we do not have a contemporary group of non-neoadjuvant-treated OeC patients. So, we were unable to assess whether LNs can show fibrosis even if there has been no neoadjuvant treatment. Assessment with certainty, whether LNs were located in or outside the RT field, was difficult in borderline cases. However, the PTV was used as uncertainty margin which compensates this partly. From 24% of the resected LNs, the exact anatomical location was not traceable as they were located in undefined fatty tissue attached to the resected specimen. However, as most were found closely to the tumour, we assume that most of them were radiated. Unfortunately, data on recurrence pattern were not available to us for analysis in the current study. Urakawa et al. have shown that in the metastatic setting, the response of the LN to chemotherapy can predict long-term survival and recurrence [22]. They found that LN non-responders presented more often with lymphatic and/or haematogenous recurrence and dissemination compared to LN responders. Results that are statistically not significant can be a consequence from the low number of participants resulting in a lack of power, future studies including more patients are needed to confirm these results.

In conclusion, this study showed a number of novel potentially clinically relevant findings. First of all, if tumour-positive LNs are found, the more tumour regression LNs show, the better the survival. Second, LNs outside the RT field can show tumour regression and even though a LN has been irradiated with the full dose, it might not show any response. Third, TRG in the LN has been shown to be related to TRG in the primary tumour. And lastly, LN-TRG within a patient can be extremely heterogenous, even LNs within the same LN station of the same patient can respond differently. Given the low incidence of LNmets outside the RT field, mainly in remote regions, this study provides no evidence for enlarging the RT field. However, further research is needed to detect LNmets more accurately during clinical staging and to better understand the heterogeneous LN-TRG seen in OeC patients.

## Electronic supplementary material

Below is the link to the electronic supplementary material.

Supplementary material 1 (DOCX 21 kb)

## Abbreviations

LN-TRG: Tumour regression in lymph nodes
nCRT: Neoadjuvant chemoradiotherapy
LNs: Lymph nodes
RT: Radiotherapy
OeC: Oesophageal cancer
TRG: Tumour regression grade
ENI: Elective lymph node irradiation
LNmets: Lymph node metastasis
LN: Lymph node
GTV: Gross tumour volume
CTV: Clinical target volume
PTV: Planning target volume
