# Supplementary material for: Lymph node response to chemoradiotherapy in oesophageal cancer patients: relationship with radiotherapy fields
Source: Esophagus. 2020 Sep 5;18(1):100–10. doi: 10.1007/s10388-020-00777-y (PMC7794105; doi:10.1007/s10388-020-00777-y)
Supplement: Supplementary file 1 — Supplementary material 1 (DOCX 21 kb) [file 10388_2020_777_MOESM1_ESM.docx]

**Table S1.** Frequency of resected lymph node (LN) stations. Numbers represent the number of patients in which respective LN stations were resected, contained at least one LN with complete regression (TRG-D LN), and contained at least one none-responding LN (ypN+).

| ***LN region*** | | **TrueN0** (n=54) | | | **Responders** (n=17) | | | **Non-complete responders** (n=46) | | | **Total** (n=117) | | |
| --- | --- | --- | --- | --- | --- | --- | --- | --- | --- | --- | --- | --- | --- |
|  |  | Resected | TRG-D LN | ypN+ | Resected | TRG-D LN | ypN+ | Resected | TRG-D LN | ypN+ | Resected | TRG-D LN | ypN+ |
| Above diaphragm | 1 | 1 | 0 | 0 | 1 | 0 | 0 | 1 | 0 | 0 | 3 | 0 | 0 |
|  | 2L | 4 | 0 | 0 | 1 | 0 | 0 | 4 | 2 | 1 | 9 | 2 | 1 |
|  | 2R | 1 | 0 | 0 | 2 | 1 | 0 | 4 | 0 | 0 | 7 | 1 | 0 |
|  | 3 | 2 | 0 | 0 | 5 | 0 | 0 | 4 | 0 | 0 | 11 | 0 | 0 |
|  | 4L | 5 | 0 | 0 | 3 | 0 | 0 | 2 | 0 | 1 | 10 | 0 | 1 |
|  | 4R | 5 | 0 | 0 | 3 | 0 | 0 | 2 | 0 | 1 | 10 | 0 | 1 |
|  | 5 | 0 | 0 | 0 | 1 | 0 | 0 | 0 | 0 | 0 | 1 | 0 | 0 |
|  | 7 | 36 | 0 | 0 | 13 | 2 | 0 | 29 | 2 | 8 | 78 | 4 | 8 |
|  | 8L | 29 | 0 | 0 | 8 | 1 | 0 | 20 | 3 | 4 | 57 | 4 | 4 |
|  | 8M | 24 | 0 | 0 | 7 | 2 | 0 | 15 | 1 | 5 | 46 | 3 | 5 |
|  | 9 | 9 | 0 | 0 | 4 | 1 | 0 | 11 | 2 | 1 | 24 | 3 | 1 |
|  | 10 | 0 | 0 | 0 | 0 | 0 | 0 | 1 | 1 | 0 | 1 | 1 | 0 |
|  | 15 | 13 | 0 | 0 | 4 | 1 | 0 | 4 | 0 | 1 | 21 | 1 | 1 |
| Below diaphragm | 16 | 20 | 0 | 0 | 6 | 1 | 0 | 12 | 0 | 4 | 38 | 1 | 4 |
|  | 17 | 29 | 0 | 0 | 10 | 5 | 0 | 31 | 6 | 19 | 70 | 11 | 19 |
|  | 18 | 33 | 0 | 0 | 10 | 3 | 0 | 33 | 2 | 11 | 76 | 5 | 11 |
|  | 19 | 30 | 0 | 0 | 8 | 1 | 0 | 26 | 3 | 11 | 64 | 4 | 11 |
|  | 20 | 19 | 0 | 0 | 12 | 4 | 0 | 19 | 3 | 9 | 50 | 7 | 9 |
|  | Unknown | 37 | 0 | 0 | 11 | 1 | 0 | 34 | 5 | 18 | 82 | 6 | 18 |

**Table S2.** Total number of lymph nodes per TRG category.

| **RT-field** | **Tumour regression grade** | | | **Total** |
| --- | --- | --- | --- | --- |
|  | B | C | D |  |
| In, n (%) | 42 (75) | 71 (68) | 80 (77) | 193 (73) |
| Out, n (%) | 4 (7) | 8 (8) | 1 (1) | 13 (5) |
| Unknown, n (%) | 10 (18) | 25 (24) | 23 (22) | 58 (22) |
| Total, n | 56 | 104 | 104 | 264 |

**Table S3.** Univariate analysis per lymph node tumour regression category: TRG-B inside RT-field (TRG-B_inRT), TRG-C inside RT-field (TRG-C_inRT), TRG-D inside RT-field (TRG-D_inRT), and TRG-B, C or D outside the RT-field (TRGBCD_outRT). Hazard ratio’s (HR) and their 95% confidence intervals (95% CI) were tested for significance using the Wald-test.

| **Category** | **Beta** | **HR (95% CI)** | **Wald-test** | **p-value** |
| --- | --- | --- | --- | --- |
| TRG-B_inRT | 0.12 | 1.1 (1-1.3) | 4.8 | 0.028 |
| TRG-C_inRT | 0.11 | 1.1 (0.96-1.3) | 2.1 | 0.15 |
| TRG-D_inRT | 0.065 | 1.1 (0.99-1.2) | 2.6 | 0.11 |
| TRG-BCD_outRT | 0.35 | 1.4 (0.81-2.4) | 1.5 | 0.22 |
